# Stakeholders’ perceptions of rehabilitation services for individuals living with disability: a survey study

**DOI:** 10.1186/s12955-016-0406-x

**Published:** 2016-01-08

**Authors:** Andrea J. Darzi, Alana Officer, Ola Abualghaib, Elie A. Akl

**Affiliations:** Department of Clinical Research Institute, American University of Beirut, Beirut, Lebanon; World Health Organization, Geneva, Switzerland; University of East Anglia, Norwich, UK; Faculty of Health Sciences, American University of Beirut, Beirut, Lebanon; Department of Internal Medicine, American University of Beirut, Beirut, Lebanon; Department of Clinical Epidemiology and Biostatistics, McMaster University, Hamilton, ON Canada; AUBMC Department of Internal Medicine, Riad-El-Solh Beirut, 1107 2020 P.O. Box: 11-0236, Beirut, Lebanon

**Keywords:** Stakeholder, Survey, Views, Perspectives, Rehabilitation, Services, Disability

## Abstract

**Background:**

The World Health Organization (WHO) was tasked with developing health system guidelines for the implementation of rehabilitation services. Stakeholders’ perceptions are an essential factor to take into account in the guideline development process.

The aim of this study was to assess stakeholders’ perceived feasibility and acceptability of eighteen rehabilitation services and the values they attach to ten rehabilitation outcomes.

**Methods:**

We disseminated an online self-administered questionnaire through a number of international and regional organizations from the different WHO regions. Eligible individuals included persons with disability, caregivers of persons with disability, health professionals, administrators and policy makers. The answer options consisted of a 9-point Likert scale.

**Results:**

Two hundred fifty three stakeholders participated. The majority of participants were health professional (64 %). In terms of outcomes, ‘Increasing access’ and ‘Optimizing utilization’ were the top service outcomes rated as critical (i.e., 7, 8 or 9 on the Likert scale) by >70 % of respondents. ‘Fewer hospital admissions’, ‘Decreased burden of care’ and ‘Increasing longevity’ were the services rated as least critical (57 %, 63 % and 58 % respectively).

In terms of services, ‘Community based rehabilitation’ and ‘Home based rehabilitation’ were found to be both definitely feasible and acceptable (75 % and 74 % respectively). ‘Integrated and decentralized rehabilitation services’ was found to be less feasible than acceptable according to stakeholders (61 % and 71 % respectively). As for ‘Task shifting’, most stakeholders did not appear to find task shifting as either definitely feasible or definitely acceptable (63 % and 64 % respectively).

**Conclusion:**

The majority of stakeholder’s perceived ‘Increasing access’ and ‘Optimizing utilization’ as most critical amongst rehabilitation outcomes. The feasibility of the ‘Integrated and decentralized rehabilitation services’ was perceived to be less than their acceptability. The majority of stakeholders found ‘Task shifting’ as neither feasible nor acceptable.

**Electronic supplementary material:**

The online version of this article (doi:10.1186/s12955-016-0406-x) contains supplementary material, which is available to authorized users.

## Background

There are over one billion people with disabilities in the world, of whom between 110-190 million experience very significant difficulties. This corresponds to about 15 % of the world’s population [1]. The prevalence of disability is growing due to population ageing and the global increase in chronic health conditions. Patterns of disability in a particular country are influenced by trends in health conditions and trends in environmental and other factors –such as road traffic crashes, natural disasters, conflict, diet and substance abuse [1].

Globally, people living with disability have poorer health, lower education achievements, less economic involvement and higher rates of poverty than people without disabilities [1]. These poor socioeconomic outcomes are largely due to the widespread barriers faced by people living with disabilities in accessing health, education, employment and information related services [2, 3]. Disability is complex and the interventions required to overcome the consequences of disability are multiple, systemic, and vary depending on context [4].

Rehabilitation is defined as “a set of measures that assist individuals, who experience or are likely to experience disability, to achieve and maintain optimum functioning in interaction with their environments” [4]. A lack of access to rehabilitation services increases the probability of disease or injury; delayed discharge; limited activities; and causes health deterioration in all aspects and a decline in the quality of life [1]. Rehabilitation is cross sectoral and may be carried out by health professionals who coordinate and collaborate with educational, employment, and financial specialists among others [1]. The provision of short and long term rehabilitation may take place in a range of settings (e.g., acute care hospitals, specialized rehabilitation wards, the community, work, or home) [5].

Rehabilitation services may be inadequate, [6–10] and there are many barriers to accessing the available services [4]. Developing policies and health systems interventions targeting the determinants of these inequalities requires urgent policy attention at national and international levels. To support the implementation of the rehabilitation aspects of the Convention on the Rights of Persons with Disabilities (CRPD), WHO was tasked with developing health services guidelines for rehabilitation services. The guidelines’ recommendations will provide guidance to stakeholders on how to improve rehabilitation services in less resourced settings in line with the recommendations in the WHO/ World Bank World report on disability [4].

WHO guidelines follow the (GRADE) methodology [11, 12]. GRADE offers a transparent and structured process for developing evidence-based guideline guidelines [13]. In addition, the methodology takes into consideration the values stakeholders assign to the outcomes of interest and their perceptions of the feasibility and acceptability of the services being recommended [14].

We conducted a survey study to assess stakeholders’ perceived feasibility and acceptability of a number rehabilitation services and the values they attach to rehabilitation outcomes.

## Methods

### Overall design

This was a stakeholder survey using a quantitative cross sectional design. We collected the primary data in June and July 2014.

### Study population

Eligible participants were those with stakes into the implementation of the planned heath services guidelines. These stakeholders included individual with physical disability, caretakers for individuals with physical disability, health professionals, administrators and policy makers. Stakeholders could represent governmental bodies, private for profit organizations, non-governmental organizations (NGOs), community based organizations (CBOs), or disabled people’s organization (DPO). These bodies could be at either the national, regional level, or international level. We excluded individuals with cognitive or mental disability, individuals younger than 18 years old, and individuals who did not speak and write the English language.

Our sampling frame consisted of a list of twelve 21 organizations including 12 organizations working in rehabilitation and “in official relations with WHO” and 9 organizations representing key international NGOs working on disability and development or regional and international DPOs representing persons with disability globally, as provided by staff from the WHO Disability and Rehabilitation Team (Additional file 1). These organizations were located in the six WHO regions. The list also included the details of the contact person of each organization. The Institutional Review Board of the American University of Beirut approved the study design. Completing the survey after reading study information was considered to be consent to participate.

### Survey questionnaire

We developed our own survey questionnaire, as we could not identify any published validated questionnaire to assess stakeholders’ perceived feasibility and acceptability of rehabilitation services and the values they attach to rehabilitation outcomes. In terms of structure and format, we based on a questionnaire to assess values and preferences developed for another WHO guidelines (Additional file 2) [15]. The content of the questionnaire was based on the interventions and outcomes that the health services guidelines for rehabilitation services were planned to address. We considered three broad constructs for the questionnaire to assess:

- Values assigned to the outcomes of the services being recommended. Outcomes of interest were are listed in Additional file 2
- Feasibility of the services being recommended (listed in Additional file 2)
- Acceptability of the services being recommended (listed in Additional file 2)

Participants rated the importance of the outcomes of interest on a ‘9’ point Likert scale with answer options ranging from 1 to 9. The scale had three anchors (not important, important, critical) for answer options 1, 5, and 9 respectively.

Participants rated feasibility and acceptability also on ‘9’ point Likert scales with answer options ranging from 1 to 9. The feasibility scale had three anchors (definitely not feasible, uncertain whether feasible or not, and definitely feasible) for answer options 1, 5 and 9. Similarly the acceptability scale had three anchors (definitely not acceptable, uncertain whether acceptable or not, and definitely acceptable) for answer options 1, 5 and 9. The survey asked participants whether they had “anything else to mention” (in the form of narrative comments) regarding each of three constructs.

The questionnaire also included a section on the following characteristics age, gender, perspective, organization, region, representation and education. We pilot tested the survey questionnaire by asking three public health practitioners to complete the survey and provide feedback about relevance and clarity.

### Data collection

Following pilot testing, we contacted all organizations on the list provided by the WHO. We emailed the contact person for each organization and asked them to forward email invitations to all members of their list serve. The invitation email included the survey link as well as a glossary (Additional file 2). The survey was hosted by LimeSurvey®. We sent out the initial invitation third week of June 2014. We then sent non-respondents two reminders at one-week intervals. Two organizations included a link to the survey on their website or in their newsletter. The invitation to participate was voluntary. Responders’ identity remained anonymous, and confidentiality was maintained.

### Data analysis

We exported survey results from Lime survey into excel sheets for reliability and consistency checks and data cleaning. For each construct of interest (values, acceptability, feasibility), we removed participants with a significant amount of missing data for that outcome. We dichotomized the three ‘construct’ variables using the two following values: favorable (values of 7, 8 and 9 on the likert scale) and unfavorable for the remaining values. We conducted descriptive analysis (numbers and valid percentages for the variables of interest using Stata (version 12). Along with the statistical data, we provide illustrative quotes taken from the participants’ narrative comments.

## Results

### Baseline characteristics

Out of 21 organizations that we contacted, 15 agreed to send invitations to their (Additional file 1). The total number of respondents was 253. We could not calculate response rate, as we did not have access to the number of members of the different listerves. Table 1 shows the baseline characteristics of respondents. Sixty five percent of respondents were from developed countries (based on the World Bank classification). The majority consisted of health professionals (64 %).

**Table 1:** Profile characteristics

Profile characteristics	n (%)
Your highest attained education degree	
Certificate or Diploma	23 (9.13 %)
Bachelors Degree	79 (31.35 %)
Masters Degree	94 (37.3 %)
Doctoral Degree	51 (20.24 %)
None of the above	5 (1.98 %)
Your Region	
Africa	15 (5.93 %)
South and South East Asia	33 (13.04 %)
Latin America	8 (3.16 %)
The Caribbean	9 (3.56 %)
North America	28 (11.07 %)
Europe	138 (54.44 %)
The Eastern Mediterranean	9 (3.56 %)
The Western Pacific	13 (5.14 %)
Your Perspective	
Persons with disability	34 (14.23 %)
User of Rehabilitation services	3 (1.26 %)
Care provider to persons with disability	9 (3.77 %)
Health Professional, Doctor	0 (0 %)
Health professional, Nurses/ midwives	0 (0 %)
Health professional, Rehabilitation personnel	125 (52.3 %)
Health professional, other	28 (11.72 %)
Policy makers	6 (2.51 %)
Health services administrators	5 (2.09 %)
Other	29 (12.13 %)
Your Main Organization	
DPO	39 (18.31 %)
CBO	22 (10.33 %)
NGO	59 (27.7 %)
Governmental organizations	73 (34.27 %)
Private for profit organization	20 (9.39 %)
Your level of responsibility	
District	87 (38.74 %)
National	70 (31.53 %)
Regional	16 (7.21 %)
International	4 (1.8 %)
Not applicable	46 (20.72 %)
Gender	
Female	178 (70.36 %)
Male	75 (29.64 %)
Age	
18 to 30	42 (16.6 %)
31 to 50	79 (31.23 %)
45 to 64	116 (45.85 %)
>64	16 (6.32 %)

**Table 2:** Assessment of values of rehabilitation outcomes (Dichotomized)

	Frequency (valid%)		Valid Total N (%)	Missing N (%)
	Not Critical	Critical		
Outcomes				
*Total Sample size (N = 176)*				
Fewer hospital admissions	75 (42.86 %)	100 (57.14 %)	175 (99.43 %)	1 (0.43 %)
Increased independence	29 (16.48 %)	147 (83.52 %)	176 (100 %)	0 (0.0 %)
Decreased burden of care	67 (38.07 %)	109 (61.93 %)	176 (100 %)	0 (0.0 %)
Return to role/occupation that is age, gender and context relevant	53 (30.11 %)	123 (69.89 %)	176 (100 %)	0 (0.0 %)
Improved Quality of life	18 (10.23 %)	158 (89.77 %)	176 (100 %)	0 (0.0 %)
Increasing longevity	73 (41.48 %)	103 (58.52 %)	176 (100 %)	0 (0.0 %)
Reducing undesired health results or complications	37 (21.02 %)	139 (78.89 %)	176 (100 %)	0 (0.0 %)
Socio-economic status (e.g., poverty)	63 (35.80 %)	113 (64.20 %)	176 (100 %)	0 (0.0 %)
Increasing access to rehabilitation services	35 (19.89 %)	141 (80.11 %)	176 (100 %)	0 (0.0 %)
Optimizing utilization of rehabilitation services	42 (23.86 %)	134 (76.14 %)	176 (100 %)	0 (0.0 %)

### Values attached to the outcomes

Two hundred thirty-one participants provided responses to their analysis. Table 2 summarizes the findings. ‘Increasing access’ and ‘optimizing utilization’ were the top service outcomes rated as critical by 80 % and 76 % of respondents respectively. ‘Fewer hospital admissions’, ‘decreased burden of care’, ‘increasing longevity’ and ‘socio-economic status’ were the services rated as least critical (57 %, 63 % and 58 % respectively).

**Table 3:** Assessment of feasibility of rehabilitation services (Dichotomized)

	Frequency (valid%)		Valid total N (%)	Missing N (%)
	Definitely not feasible	Definitely feasible		
Rehabilitation Services				
*Total N (%) = 176 (100 %)*				
The use of questionnaire for identifying rehabilitation needs (relative to no such use)	85 (48.57 %)	90 (51.43 %)	175 (99.43 %)	1 (0.57 %)
Integrated and decentralized rehabilitation services (relative to centralized rehabilitation services)	69 (39.43 %)	106 (60.57 %)	175 (99.43 %)	1 (0.57 %)
Rehabilitation services funded by both public and private sector (relative to those only publicly funded or only privately funded)	82 (46.86 %)	93 (53.14 %)	175 (99.43 %)	1 (0.57 %)
Rehabilitation services that provide free care or subsidized care for the poor (relative to no such care)	52 (29.71 %)	123 (70.29 %)	175 (99.43 %)	1 (0.57 %)
Health insurance coverage for rehabilitation services (relative to no health insurance coverage)	80 (45.71 %)	95 (54.29 %)	175 (99.43 %)	1 (0.57 %)
Providing rehabilitation services within specialized hospitals and units (relative to general hospitals or non specialized units)	58 (33.14 %)	117 (66.86 %)	175 (99.43 %)	1 (0.57 %)
Having rehabilitation delivered through your health provider (relative to having rehabilitation delivered through other providers /services like social welfare.	67 (38.51 %)	107 (61.49 %)	174 (98.86 %)	2 (1.14 %)
Community based rehabilitation (relative to hospital or clinic based rehabilitation)	44 (25.14 %)	131 (74.86 %)	175 (99.43 %)	1 (0.57 %)
Multidisciplinary rehabilitation integrated within trauma care (relative to trauma care without rehabilitation services)	47 (26.86 %)	128 (73.14 %)	175 (99.43 %)	1 (0.57 %)
The use of data collection / management and dissemination systems (relative to no such use)	64 (36.57 %)	111 (63.43 %)	175 (99.43 %)	1 (0.57 %)
Increasing the culture of data collection and use as well as acceptability and reliability of data (relatively to not increasing such a culture)	59 (33.71 %)	116 (66.29 %)	175 (99.43 %)	1 (0.57 %)
Provision of assistive technology free of charge (relative to prescription only)	52 (29.89 %)	122 (70.11 %)	174 (98.86 %)	2 (1.14 %)
Educational intervention promoting the use of assistive technology (relative to no such intervention)	35 (20.11 %)	139 (79.89 %)	174 (98.86 %)	2 (1.14 %)
Tele audiology in comparison (relative to standard face-to-face audiology)	104 (59.77 %)	70 (40.23 %)	174 (98.86 %)	2 (1.14 %)
Engaging clinicians / managers to collect and use data (relative to no such engagement)	69 (39.43 %)	106 (60.57 %)	175 (99.43 %)	1 (0.57 %)
Home-based rehabilitation programs (relative to usual care)	46 (26.29 %)	129 (73.71 %)	175 (99.43 %)	1 (0.57 %)
Tele rehabilitation strategies (relative to usual care)	86 (49.14 %)	89 (50.86 %)	175 (99.43 %)	1 (0.57 %)
Task-shifting (relative to usual care)	109 (62.64 %)	65 (37.36 %)	174 (98.86 %)	2 (1.14 %)

### Perceived feasibility and acceptability

One hundred eighty-one 181 participants provided responses to allow their inclusion in this analysis. Table 3 and 4 summarizes the findings. Most stakeholders perceived ‘community based rehabilitation’ and ‘home based rehabilitation’ as interventions that are both definitely feasible (75 % and 74 % respectively) and acceptable (79 % and 82 % respectively). The results showed variability in the perception of feasibility and acceptability in relation to ‘integrated and decentralized rehabilitation services’ (61 % and 71 % respectively). 37 % and 36 % respectively of stakeholders rated ‘task shifting’ as definitely feasible and definitely acceptable.

**Table 4:** Assessment of acceptability of rehabilitation services (Dichotomized)

	Frequency (valid%)		Valid total N (%)	Missing N (%)
	Definitely not acceptable	Definitely acceptable		
Rehabilitation Services				
*Total N (%) = 176 (100 %)*				
The use of questionnaire for identifying rehabilitation needs (relative to no such use)	72 (41.62 %)	101 (58.38 %)	173 (98.30 %)	3 (1.70 %)
Integrated and decentralized rehabilitation services (relative to centralized rehabilitation services)	50 (28.90 %)	123 (71.10 %)	173 (98.30 %)	3 (1.70 %)
Rehabilitation services funded by both public and private sector (relative to those only publicly funded or only privately funded)	63 (36.42 %)	110 (63.58 %)	173 (98.30 %)	3 (1.70 %)
Rehabilitation services that provide free care or subsidized care for the poor (relative to no such care)	39 (22.54 %)	134 (77.46 %)	173 (98.30 %)	3 (1.70 %)
Health insurance coverage for rehabilitation services (relative to no health insurance coverage)	67 (38.73 %)	106 (61.27 %)	173 (98.30 %)	3 (1.70 %)
Providing rehabilitation services within specialized hospitals and units (relative to general hospitals or non specialized units)	55 (31.79 %)	118 (68.21 %)	173 (98.30 %)	3 (1.70 %)
Having rehabilitation delivered through your health provider (relative to having rehabilitation delivered through other providers /services like social welfare.	46 (26.59 %)	127 (73.41 %)	173 (98.30 %)	3 (1.70 %)
Community based rehabilitation (relative to hospital or clinic based rehabilitation)	36 (20.81 %)	137 (79.19 %)	173 (98.30 %)	3 (1.70 %)
Multidisciplinary rehabilitation integrated within trauma care (relative to trauma care without rehabilitation services)	41 (23.70 %)	132 (76.30 %)	173 (98.30 %)	3 (1.70 %)
The use of data collection / management and dissemination systems (relative to no such use)	54 (31.03 %)	120 (68.97 %)	174 (98.86 %)	2 (1.14 %)
Increasing the culture of data collection and use as well as acceptability and reliability of data (relatively to not increasing such a culture)	52 (29.89 %)	122 (70.11 %)	174 (98.86 %)	2 (1.14 %)
Provision of assistive technology free of charge (relative to prescription only)	52 (29.89 %)	122 (70.11 %)	174 (98.86 %)	2 (1.14 %)
Educational intervention promoting the use of assistive technology (relative to no such intervention)	35 (20.22 %)	139 (79.89 %)	174 (98.86 %)	2 (1.14 %)
Tele audiology in comparison (relative to standard face-to-face audiology)	104 (59.77 %)	70 (40.23 %)	174 (98.86 %)	2 (1.14 %)
Engaging clinicians / managers to collect and use data (relative to no such engagement)	57 (32.76 %)	117 (67.24 %)	174 (98.86 %)	2 (1.14 %)
Home-based rehabilitation programs (relative to usual care)	31 (17.82 %)	143 (82.18 %)	174 (98.86 %)	2 (1.14 %)
Tele rehabilitation strategies (relative to usual care)	89 (51.15 %)	85 (48.85 %)	174 (98.86 %)	2 (1.14 %)
Task-shifting (relative to usual care)	112 (64.37 %)	62 (35.63 %)	174 (98.86 %)	2 (1.14 %)

### Findings from narrative comments

As for the narrative comments, the participants’ noted the importance of patient centered care and user involvement for achieving optimal outcomes. A participating rehabilitation highlighted the need for “increased social interaction and communication” while another participant also with the perspective of perspective of rehabilitation personnel noted that “flexibility with a person centered approach is crucial rather than a blanket policy that all must ‘fit in to’.” Also a participant with the same perspective commented that: “I would like to see more people with disabilities involved in the decision making and collecting of data; more home and community based rehabilitation that is not doctor driven.”

In terms of feasibility, one participant with the perspective of a health professional stated that “Rehab provided by a national health service contributed to by all taxpayers to ensure free access to rehab is feasible, just need political will and popular support.” Another participant with the perspective of rehabilitation personnel stated that: “In many cases feasibility is tightly linked to staffing levels; often we have the technology and provision for more person centered treatment but not the staff to implement it e.g. home based rehab.” In terms of acceptability, one participant with the same perspective highlighted that “the rehabilitation pathway should enable flexibility and movement between service providers at different points in a patient’s condition.”

## Discussion

In summary, we conducted an international survey of stakeholders’ perceptions of rehabilitation services for individuals living with disability. The majority of respondents was from the developed countries and perceived ‘increasing access’ and ‘optimizing utilization’ as most critical amongst rehabilitation outcomes. ‘Fewer hospital admissions’, ‘decreased burden of care’, ‘increasing longevity’ and ‘socio-economic status’ were the services rated as least critical. Most stakeholders perceived ‘community based rehabilitation’ and ‘home based rehabilitation’ as interventions that are both definitely feasible and acceptable. The results showed variability in the perception of feasibility and acceptability in relation to ‘integrated and decentralized rehabilitation services’. The majority of stakeholders found ‘task shifting’ to be neither feasible nor acceptable.

The study has a number of limitations. As the study sample consisted mostly of health professionals, the generalizability of our findings to people living with disability, carers of those people and policymakers may be limited. Indeed, these different subgroups are likely to have different perspectives, but the relatively limited numbers of participants did not allow a meaningful subgroup analysis. Similarly, the fact that 65 % of respondents were from developed countries may limit the generalizability to developing countries.

We were unable to quantify the number of invitations sent (and thus calculate the sample size or response rate) as our only contact was with the contact persons of organizations. Although a standardized tool was not available, we developed this survey questionnaire based on a stakeholder survey developed for another WHO guideline on ‘transformative up scaling of health professional education’ [15]. The lack of a formal process to ensure the validity and reliability of the questionnaire does represent a limitation of this study.

Despite these limitations, the wide array of views and perspectives of stakeholders measured by this study adds value to the process of guideline development. This is particularly relevant when considering that stakeholders’ perspectives are generally assessed through the input of a limited number of panelists participating in the guideline process [16]. This study contributes to the currently scarce empirical evidence on the perceptions of stakeholders relating to feasibility and acceptability of different rehabilitation services and the values assigned to the service outcomes for people living with disability. Indeed, while we identified a number of studies assessing the acceptability and feasibility of clinical rehabilitation interventions [17], we did not identify any for health services interventions.

A primary feature of any policy design process is the assurance of involving members who have a stake or vested interest in the outcome of that policy and are the intended users who can most directly benefit from it, as well as others who have a direct or indirect interest in its implementation. Engaging stakeholders in such process enhances intended users’ understanding and acceptance of the utility of designed policies. Stakeholders are much more likely to buy into and support if they are involved in the process from the beginning. The importance of Engaging stakeholders (mainly DPOs) in decision making process related to disability policies has been confirmed by the guiding principles of the UN convention on rights of persons with disabilities that was adopted in 2008 and have been ratified by 154 . Interpreting such principles into feasible methods requires further research to validate scales for measurement of the constructs of values, feasibility and acceptability, including the one we used in this study. Also, there is a need to identify and evaluate processes to integrate findings of stakeholder surveys in the decision making process of guideline development.

## Conclusion

Our international survey found a wide variability in the perceptions of stakeholders’ of the feasibility and acceptability of rehabilitation services for individuals living with disability, and in their perceptions of the outcomes relevant to those services. Generally, the majority of participants perceived ‘community based rehabilitation’ and ‘home based rehabilitation’ to be feasible and acceptable. Similarly, the majority of participants perceived ‘increasing access’ and ‘optimizing utilization’ as the most critical amongst rehabilitation outcomes.

## Additional files

Additional file 1:
**List of organizations working in rehabilitation in official relations with WHO.** (DOC 168 kb) Additional file 2:
**Survey and glossary.** (DOC 127 kb)

## Abbreviations

WHO: World Health Organization
CRPD: Convention on the rights of persons with disabilities
GRADE: Grading of recommendations assessment, development and evaluation
NGO: Non-Governmental organization
CBO: Community- based organization
DPO: Disability people’s organization
UN: United Nations
