# Supplementary material for: Stakeholders’ perceptions of rehabilitation services for individuals living with disability: a survey study
Source: Health Qual Life Outcomes. 2016 Jan 8;14:2. doi: 10.1186/s12955-016-0406-x (PMC4706719; doi:10.1186/s12955-016-0406-x)
Supplement: Additional file 1: — List of organizations working in rehabilitation in official relations with WHO. (DOC 168 kb) [file 12955_2016_406_MOESM1_ESM.doc]

**Appendix 2:** **List of organizations working in rehabilitation in official relations with WHO**

| **Organizations name** | **First name** | **Surname** |  | **Email** |
| --- | --- | --- | --- | --- |
| **Rehabilitation International (RI)** | Jan Arne | Monsbakken | President | [jam@sanitetskvinnene.no](mailto:jam@sanitetskvinnene.no) |
|  |  |  | Rehabilitation International |  |
|  |  |  | 25 E. 21st Street, 4th Floor |  |
|  |  |  | New York, NY 10010 |  |
|  |  |  | Etats-Unis d'Amérique |  |
| **World Confederation for Physical Therapy (WCPT)** | Catherine | Sykes | WHO Focal person | [csykes@wcpt.org](mailto:csykes@wcpt.org) |
| **World Federation of Occupational Therapists (WFOT)** | Marilyn | Pattison | Executive Director | [Marilyn Pattison <marilyn@mpot.com.au>](mailto:sharon.brintnell@ualberta.ca) |
|  |  |  | World Federation of Occupational Therapists |  |
|  |  |  | c/o Dept. of Occupational Therapy |  |
|  |  |  | Faculty of Rehabilitation Medicine, University of Alberta |  |
|  |  |  | 2-64 Corbett Hall |  |
|  |  |  | Edmonton, T6G 2G4, Alberta |  |
|  |  |  | Canada |  |
| **International Society of Physical and Rehabilitation Medicine (ISPRM)** | Gerold | Stucki | President-Elect | [gerold.stucki@paranet.ch](mailto:gerold.stucki@paranet.ch) |
| **International Society for Prosthetics and Orthotics (ISPO)** | Carson | Harte | WHO focal person | [Carson@cambodiatrust.org.uk](mailto:Carson@cambodiatrust.org.uk) |
|  |  |  | 22-24 Rue du Luxembourg |  |
|  |  |  | B-1000 Brussels |  |
| **International Association of Logopedics and Phoniatrics (IALP)** | Tanya | Gallagher | President | [tmgallag@illinois.edu](mailto:tmgallag@illinois.edu) |
|  |  |  | Tilweg 1 |  |
|  |  |  | Ulrum |  |
|  |  |  | The Netherlands |  |
|  |  |  | 9971 CW |  |
| **International Federation of Hard of Hearing (IFHOH)** | Ruth | Warick | President | [ruth.warick@ubc.ca](mailto:ruth.warick@ubc.ca) |
|  |  |  | Box 6605 |  |
|  |  |  | Stockholm |  |
|  |  |  | S-113 84 |  |
| **World Blind Union (WBU)** | Arnt | Holte | WHO Focal person | [arnt.holte@blindeforbundet.no](mailto:arnt.holte@blindeforbundet.no) |
| **WFD** | C. | Allen | WFD Secretariat | [wfdpresident@gmail.com](mailto:wfdpresident@gmail.com) |
|  |  |  | PO Box 65 |  |
|  |  |  | FIN-00401 |  |
|  |  |  | Helsinki, Finland |  |
| **WFD** | Meri | Hyrske-Fischer |  | [Meri.Hyrske-Fischer@kl-deaf.fi](mailto:Meri.Hyrske-Fischer@kl-deaf.fi) |
| **CBM** | Karen | Heinicke-Motsch |  | [kheinickemotsch@cbmus.org](mailto:kheinickemotsch@cbmus.org) |
| **Inclusion International (II)** | Klaus | Lachwitz | President | [info@inclusion-international.org](mailto:info@inclusion-international.org) |
| **International Association for the Prevention of Blindness (IAPB)** | Peter | Ackland | Chief Executive Officer | [packland@iapb.org](mailto:packland@iapb.org) |
| **DPI** | Javed | Abidi | Chairperson | [secretariat@ncpedp.org](mailto:secretariat@ncpedp.org) |
| **World Federation of the DeafBlind** | Geir | Jensen | Secretary General | [geir.jensen@fndb.no](mailto:geir.jensen@fndb.no) |
| **Pacific Islands Forum** | Fred | Miller | Disability Coordination Officer | [frederickm@forumsec.org.fj](mailto:frederickm@forumsec.org.fj) |
| **International Disability Alliance (IDA)** | Ellen | Walker | WHO Focal person | ewalker@ida-secretariat.org |
| **AOPD (Arab Organization for People with disabilities** | Jahda | Abu Khalil | Executive Director | [aodp@cyberia.net.lb](mailto:aodp@cyberia.net.lb) |
| **EDF (European Disability Forum )** | Javier | Guemes | Director | [javier.guemes@edf-feph.org](mailto:javier.guemes@edf-feph.org) |
| **Mobility India North East - Inclusive Development Centre** | Sunyl | Deepak | Head of Mobility India North East - Inclusive Development Centre | [sunil.deepak@gmail.com](mailto:sunil.deepak@gmail.com) |
| **Royal Cornwall Hospitals NHS Trust and Bone and Joint Decade (BJD) International Co-ordinating Council** | Anthony | Woolf | Professorat Royal Cornwall Hospitals NHS and chair of Bone and Joint Decade (BJD) International Co-ordinating Council | [anthony.woolf@btinternet.com](mailto:anthony.woolf@btinternet.com) |

**Tables (Descriptive Statistics)**

***Table 1: Profile characteristics***

| **Profile characteristics** | **n (%)** |
| --- | --- |
| **Your highest attained education degree** |  |
| Certificate or Diploma | 23 (9.13%) |
| Bachelors Degree | 79 (31.35%) |
| Masters Degree | 94 (37.3%) |
| Doctoral Degree | 51 (20.24%) |
| None of the above | 5 (1.98%) |
| **Your Region** |  |
| Africa | 15 (5.93%) |
| South and South East Asia | 33 (13.04%) |
| Latin America | 8 (3.16%) |
| The Caribbean | 9 (3.56%) |
| North America | 28 (11.07%) |
| Europe | 138 (54.44%) |
| The Eastern Mediterranean | 9 (3.56%) |
| The Western Pacific | 13 (5.14%) |
| **Your Perspective** |  |
| Persons with disability | 34 (14.23%) |
| User of Rehabilitation services | 3 (1.26%) |
| Care provider to persons with disability | 9 (3.77%) |
| Health Professional, Doctor | 0 (0%) |
| Health professional, Nurses/ midwives | 0 (0%) |
| Health professional, Rehabilitation personnel | 125 (52.3%) |
| Health professional, other | 28 (11.72%) |
| Policy makers | 6 (2.51%) |
| Health services administrators | 5 (2.09%) |
| Other | 29 (12.13%) |
| **Your Main Organization** |  |
| DPO | 39 (18.31%) |
| CBO | 22 (10.33%) |
| NGO | 59 (27.7%) |
| Governmental organizations | 73 (34.27%) |
| Private for profit organization | 20 (9.39%) |
| **Your level of responsibility** |  |
| District | 87 (38.74%) |
| National | 70 (31.53%) |
| Regional | 16 (7.21%) |
| International | 4 (1.8%) |
| Not applicable | 46 (20.72%) |
| **Gender** |  |
| Female | 178 (70.36%) |
| Male | 75 (29.64%) |
| **Age** |  |
| 18 to 30 | 42 (16.6%) |
| 31 to 50 | 79 (31.23%) |
| 45 to 64 | 116 (45.85%) |
| >64 | 16 (6.32%) |

*Table 2: Assessment of Values of Rehabilitation Outcomes (Dichotomized)*

|  | **Frequency (valid%)** | | **Valid Total N (%)** | **Missing N (%)** |
| --- | --- | --- | --- | --- |
|  | **Not Critical** | **Critical** |  |  |
| **Outcomes** |  |  |  |  |
| *Total Sample size (N=176)* |  |  |  |  |
| Fewer hospital admissions | 75 (42.86%) | 100 (57.14%) | 175 (99.43%) | 1 (0.43%) |
| Increased independence | 29 (16.48%) | 147 (83.52%) | 176 (100%) | 0 (0.0%) |
| Decreased burden of care | 67 (38.07%) | 109 (61.93%) | 176 (100%) | 0 (0.0%) |
| Return to role/occupation that is age, gender and context relevant | 53 (30.11%) | 123 (69.89%) | 176 (100%) | 0 (0.0%) |
| Improved Quality of life | 18 (10.23%) | 158 (89.77%) | 176 (100%) | 0 (0.0%) |
| Increasing longevity | 73 (41.48%) | 103 (58.52%) | 176 (100%) | 0 (0.0%) |
| Reducing undesired health results or complications | 37 (21.02%) | 139 (78.89%) | 176 (100%) | 0 (0.0%) |
| Socio-economic status (e.g., poverty) | 63 (35.80%) | 113 (64.20%) | 176 (100%) | 0 (0.0%) |
| Increasing access to rehabilitation services | 35 (19.89%) | 141 (80.11%) | 176 (100%) | 0 (0.0%) |
| Optimizing utilization of rehabilitation services | 42 (23.86%) | 134 (76.14%) | 176 (100%) | 0 (0.0%) |

*Table 3: Assessment of Feasibility of Rehabilitation Services (Dichotomized)*

|  | **Frequency (valid%)** | | **Valid Total N (%)** | **Missing N (%)** |
| --- | --- | --- | --- | --- |
|  | **Definitely Not feasible** | **Definitely Feasible** |  |  |
| **Rehabilitation Services** |  |  |  |  |
| *Total N (%)= 176 (100%)* |  |  |  |  |
| The use of questionnaire for identifying rehabilitation needs (relative to no such use) | 85 (48.57%) | 90 (51.43%) | 175 (99.43%) | 1 (0.57%) |
| Integrated and decentralized rehabilitation services (relative to centralized rehabilitation services) | 69 (39.43%) | 106 (60.57%) | 175 (99.43%) | 1 (0.57%) |
| Rehabilitation services funded by both public and private sector (relative to those only publicly funded or only privately funded) | 82 (46.86%) | 93 (53.14%) | 175 (99.43%) | 1 (0.57%) |
| Rehabilitation services that provide free care or subsidized care for the poor (relative to no such care) | 52 (29.71%) | 123 (70.29%) | 175 (99.43%) | 1 (0.57%) |
| Health insurance coverage for rehabilitation services (relative to no health insurance coverage) | 80 (45.71%) | 95 (54.29%) | 175 (99.43%) | 1 (0.57%) |
| Providing rehabilitation services within specialized hospitals and units (relative to general hospitals or non specialized units) | 58 (33.14%) | 117 (66.86%) | 175 (99.43%) | 1 (0.57%) |
| Having rehabilitation delivered through your health provider (relative to having rehabilitation delivered through other providers /services like social welfare. | 67 (38.51%) | 107 (61.49%) | 174 (98.86%) | 2 (1.14%) |
| Community based rehabilitation (relative to hospital or clinic based rehabilitation) | 44 (25.14%) | 131 (74.86%) | 175 (99.43%) | 1 (0.57%) |
| Multidisciplinary rehabilitation integrated within trauma care (relative to trauma care without rehabilitation services) | 47 (26.86%) | 128 (73.14%) | 175 (99.43%) | 1 (0.57%) |
| The use of data collection / management and dissemination systems (relative to no such use) | 64 (36.57%) | 111 (63.43%) | 175 (99.43%) | 1 (0.57%) |
| Increasing the culture of data collection and use as well as acceptability and reliability of data (relatively to not increasing such a culture) | 59 (33.71%) | 116 (66.29%) | 175 (99.43%) | 1 (0.57%) |
| Provision of assistive technology free of charge (relative to prescription only) | 52 (29.89%) | 122 (70.11%) | 174 (98.86%) | 2 (1.14%) |
| Educational intervention promoting the use of assistive technology (relative to no such intervention) | 35 (20.11%) | 139 (79.89%) | 174 (98.86%) | 2 (1.14%) |
| Tele audiology in comparison (relative to standard face-to-face audiology) | 104 (59.77%) | 70 (40.23%) | 174 (98.86%) | 2 (1.14%) |
| Engaging clinicians / managers to collect and use data (relative to no such engagement) | 69 (39.43%) | 106 (60.57%) | 175 (99.43%) | 1 (0.57%) |
| Home-based rehabilitation programs (relative to usual care) | 46 (26.29%) | 129 (73.71%) | 175 (99.43%) | 1 (0.57%) |
| Tele rehabilitation strategies (relative to usual care) | 86 (49.14%) | 89 (50.86%) | 175 (99.43%) | 1 (0.57%) |
| Task-shifting (relative to usual care) | 109 (62.64%) | 65 (37.36%) | 174 (98.86%) | 2 (1.14%) |

*Table 4: Assessment of Acceptability of Rehabilitation Services (Dichotomized)*

|  | **Frequency (valid%)** | | **Valid Total N (%)** | **Missing N (%)** |
| --- | --- | --- | --- | --- |
|  | **Definitely Not Acceptable** | **Definitely Acceptable** |  |  |
| **Rehabilitation Services** |  |  |  |  |
| *Total N (%)= 176 (100%)* |  |  |  |  |
| The use of questionnaire for identifying rehabilitation needs (relative to no such use) | 72 (41.62%) | 101 (58.38%) | 173 (98.30%) | 3 (1.70%) |
| Integrated and decentralized rehabilitation services (relative to centralized rehabilitation services) | 50 (28.90%) | 123 (71.10%) | 173 (98.30%) | 3 (1.70%) |
| Rehabilitation services funded by both public and private sector (relative to those only publicly funded or only privately funded) | 63 (36.42%) | 110 (63.58%) | 173 (98.30%) | 3 (1.70%) |
| Rehabilitation services that provide free care or subsidized care for the poor (relative to no such care) | 39 (22.54%) | 134 (77.46%) | 173 (98.30%) | 3 (1.70%) |
| Health insurance coverage for rehabilitation services (relative to no health insurance coverage) | 67 (38.73%) | 106 (61.27%) | 173 (98.30%) | 3 (1.70%) |
| Providing rehabilitation services within specialized hospitals and units (relative to general hospitals or non specialized units) | 55 (31.79%) | 118 (68.21%) | 173 (98.30%) | 3 (1.70%) |
| Having rehabilitation delivered through your health provider (relative to having rehabilitation delivered through other providers /services like social welfare. | 46 (26.59%) | 127 (73.41%) | 173 (98.30%) | 3 (1.70%) |
| Community based rehabilitation (relative to hospital or clinic based rehabilitation) | 36 (20.81%) | 137 (79.19%) | 173 (98.30%) | 3 (1.70%) |
| Multidisciplinary rehabilitation integrated within trauma care (relative to trauma care without rehabilitation services) | 41 (23.70%) | 132 (76.30%) | 173 (98.30%) | 3 (1.70%) |
| The use of data collection / management and dissemination systems (relative to no such use) | 54 (31.03%) | 120 (68.97%) | 174 (98.86%) | 2 (1.14%) |
| Increasing the culture of data collection and use as well as acceptability and reliability of data (relatively to not increasing such a culture) | 52 (29.89%) | 122 (70.11%) | 174 (98.86%) | 2 (1.14%) |
| Provision of assistive technology free of charge (relative to prescription only) | 52 (29.89%) | 122 (70.11%) | 174 (98.86%) | 2 (1.14%) |
| Educational intervention promoting the use of assistive technology (relative to no such intervention) | 35 (20.22%) | 139 (79.89%) | 174 (98.86%) | 2 (1.14%) |
| Tele audiology in comparison (relative to standard face-to-face audiology) | 104 (59.77%) | 70 (40.23%) | 174 (98.86%) | 2 (1.14%) |
| Engaging clinicians / managers to collect and use data (relative to no such engagement) | 57 (32.76%) | 117 (67.24%) | 174 (98.86%) | 2 (1.14%) |
| Home-based rehabilitation programs (relative to usual care) | 31 (17.82%) | 143 (82.18%) | 174 (98.86%) | 2 (1.14%) |
| Tele rehabilitation strategies (relative to usual care) | 89 (51.15%) | 85 (48.85%) | 174 (98.86%) | 2 (1.14%) |
| Task-shifting (relative to usual care) | 112 (64.37%) | 62 (35.63%) | 174 (98.86%) | 2 (1.14%) |
