# Supplementary material for: Stakeholders’ perceptions of rehabilitation services for individuals living with disability: a survey study
Source: Health Qual Life Outcomes. 2016 Jan 8;14:2. doi: 10.1186/s12955-016-0406-x (PMC4706719; doi:10.1186/s12955-016-0406-x)
Supplement: Additional file 2: — Survey and glossary. (DOC 127 kb) [file 12955_2016_406_MOESM2_ESM.doc]

**Appendices:**

**Appendix 1: Survey and Glossary**

**Policy guidelines on rehabilitation services for individuals living with disability - Assessment of views and perspectives of stakeholders**

***INTRODUCTION***

The World Health Organization (WHO) is developing policy guidelines for rehabilitation services for individuals living with disabilities. We are conducting the present survey to better understand the views of stakeholders in relation to the feasibility and acceptability for those services, and the values assigned to the outcomes of the services.

Participation in our survey is voluntary; you have the right to refuse to respond to any or all questions, but we request that you kindly fill out all questions as far as possible in order to help us to have relevant and helpful recommendations. We will consider your consent is given when you choose to take the survey and “pressing NEXT” as recognition of your voluntary participation. Please be assured that your responses will remain confidential, i.e. information you provide will be used only in generalized descriptions. Completion of the questionnaire should take approximately 5 to 10 minutes and you will be able to save your answers as you go along, or return to them at a later time. Please complete the ratings from you own perspective, taking into account your specific/local context.

Should you encounter any questions on which you would like further clarification or guidance, please feel free to consult with (*Dr. Elie Akl*), our regional/country contact point for this survey.

You can find descriptions of terms marked with an asterisk (*) in the accompanying glossary.

1. **Your profile**

**Your highest attained educational degree**

- Certificate or diploma
- Bachelor's degree (e.g. BSc)
- Master's degree (e.g. MSc, MA)
- Doctoral degree (MD, PhD or equivalent)
- None of the above

**Your Region:**

- Africa
- South and South East Asia
- Latin America
- The Caribbean
- North America
- Europe
- The Eastern Mediterranean
- The Western Pacific

**Country** ________________________

**You perspective would represent best that of (choose one):**

- Persons with disability
- User of rehabilitation services
- Care provider to persons with disability
- Health Professionals:
- Doctors
- Nurses/midwives
- Rehabilitation personnel
- Other ________________________
- Policy makers
- Health services administrators
- Other________________________

**Your main organization (choose one):**

- Disabled people’s organization (DPO)
- Community Based organization (CBO)
- Non governmental organization (NGO)
- Governmental organization
- Private for profit organization
- Other: ________

**Your level of responsibility (if applicable)**

- District (within a country)
- National (at the country level)
- Regional (at a level broader than national)
- International
- Not applicable

**Your gender:**

- Female
- Male

**Your age:**

- 18−30
- 31−50
- 51−64
- >6

**Assessment of rehabilitation outcomes**

While all outcomes of rehabilitation are important, people’s perceptions of their importance may vary. Please rate the importance below from your perspective

**How important are the following outcomes to people living with disability:**

**Assessment of rehabilitation outcomes**

While all outcomes of rehabilitation are important, people’s perceptions of their importance may vary. Please rate the importance below from your perspective

**How important are the following outcomes to people living with disability:**

|  | **Not important** |  |  |  | **Important** |  |  |  | **Critical** |  |
| --- | --- | --- | --- | --- | --- | --- | --- | --- | --- | --- |
| **Fewer hospital admissions** | **1** | 2 | 3 | 4 | **5** | 6 | 7 | 8 | **9** |  |
| **Increased independence** | **1** | 2 | 3 | 4 | **5** | 6 | 7 | 8 | **9** |  |
| **Return to role/occupation that is age, gender and context relevant** | **1** | 2 | 3 | 4 | **5** | 6 | 7 | 8 | **9** |  |
| **Decreased burden of care** | **1** | 2 | 3 | 4 | **5** | 6 | 7 | 8 | **9** |  |
| **Improved Quality of life** | **1** | 2 | 3 | 4 | **5** | 6 | 7 | 8 | **9** |  |
| **Increasing longevity** | **1** | 2 | 3 | 4 | **5** | 6 | 7 | 8 | **9** |  |
| **Reducing undesired health results or complications** | **1** | 2 | 3 | 4 | **5** | 6 | 7 | 8 | **9** |  |
| **Socio-economic status (e.g., poverty)** | **1** | 2 | 3 | 4 | **5** | 6 | 7 | 8 | **9** |  |
| **Increasing access to rehabilitation services** | **1** | 2 | 3 | 4 | **5** | 6 | 7 | 8 | **9** |  |
| **Optimizing utilization of rehabilitation services** | **1** | 2 | 3 | 4 | **5** | 6 | 7 | 8 | **9** |  |

**Assessment of rehabilitation interventions**

**Please rate the FEASIBILITY of the following rehabilitation services; please assess feasibility relative to the services described in brackets, when described**

|  | **Definitely not feasible** |  |  |  | **Uncertain whether feasible or not** |  |  |  | **Definitely feasible** |  |
| --- | --- | --- | --- | --- | --- | --- | --- | --- | --- | --- |
| **The use of questionnaire for identifying rehabilitation needs (relative to no such use)** | **1** | 2 | 3 | 4 | **5** | 6 | 7 | 8 | **9** |  |
| **Integrated and decentralized rehabilitation services (relative to centralized rehabilitation services)** | **1** | 2 | 3 | 4 | **5** | 6 | 7 | 8 | **9** |  |
| **Rehabilitation services funded by both public and private sector (relative to those only publicly funded or only privately funded)** | **1** | 2 | 3 | 4 | **5** | 6 | 7 | 8 | **9** |  |
| **Rehabilitation services that provide free care or subsidized care for the poor (relative to no such care)** | **1** | 2 | 3 | 4 | **5** | 6 | 7 | 8 | **9** |  |
| **Health insurance coverage for rehabilitation services (relative to no health insurance coverage)** | **1** | 2 | 3 | 4 | **5** | 6 | 7 | 8 | **9** |  |
| **Providing rehabilitation services within specialized hospitals and units (relative to general hospitals or non specialized units)** | **1** | 2 | 3 | 4 | **5** | 6 | 7 | 8 | **9** |  |
| **Having rehabilitation delivered through your health provider (relative to having rehabilitation delivered through other providers /services like social welfare.** | **1** | 2 | 3 | 4 | **5** | 6 | 7 | 8 | **9** |  |
| **Community based rehabilitation (relative to hospital or clinic based rehabilitation)** | **1** | 2 | 3 | 4 | **5** | 6 | 7 | 8 | **9** |  |
| **Multidisciplinary rehabilitation integrated within trauma care (relative to trauma care without rehabilitation services)** | **1** | 2 | 3 | 4 | **5** | 6 | 7 | 8 | **9** |  |
| **The use of data collection / management and dissemination systems (relative to no such use)** | **1** | 2 | 3 | 4 | **5** | 6 | 7 | 8 | **9** |  |
| **Increasing the culture of data collection and use as well as acceptability and reliability of data (relatively to not increasing such a culture)** | **1** | 2 | 3 | 4 | **5** | 6 | 7 | 8 | **9** |  |
| **Provision of assistive technology free of charge (relative to prescription only)** | **1** | 2 | 3 | 4 | **5** | 6 | 7 | 8 | **9** |  |
| **Educational intervention promoting the use of assistive technology (relative to no such intervention)** | **1** | 2 | 3 | 4 | **5** | 6 | 7 | 8 | **9** |  |
| **Tele audiology in comparison (relative to standard face-to-face audiology)** | **1** | 2 | 3 | 4 | **5** | 6 | 7 | 8 | **9** |  |
| **Engaging clinicians / managers to collect and use data (relative to no such engagement)** | **1** | 2 | 3 | 4 | **5** | 6 | 7 | 8 | **9** |  |
| **Home-based rehabilitation programs (relative to usual care)** | **1** | 2 | 3 | 4 | **5** | 6 | 7 | 8 | **9** |  |
| **Tele rehabilitation strategies (relative to usual care)** | **1** | 2 | 3 | 4 | **5** | 6 | 7 | 8 | **9** |  |
| **Task-shifting (relative to usual care)** | **1** | 2 | 3 | 4 | **5** | 6 | 7 | 8 | **9** |  |

**Please rate the ACCEPTABILITY of the following rehabilitation interventions; please assess the acceptability relative to the services described in brackets, when described**

|  | **Definitely not acceptable** |  |  |  | **Uncertain whether acceptable or not** |  |  |  | **Definitely acceptable** |  |
| --- | --- | --- | --- | --- | --- | --- | --- | --- | --- | --- |
| **The use of questionnaire for identifying rehabilitation needs (relative to no such use)** | **1** | 2 | 3 | 4 | **5** | 6 | 7 | 8 | **9** |  |
| **Integrated and decentralized rehabilitation services (relative to centralized rehabilitation services)** | **1** | 2 | 3 | 4 | **5** | 6 | 7 | 8 | **9** |  |
| **Rehabilitation services funded by both public and private sector (relative to those only publicly funded or only privately funded)** | **1** | 2 | 3 | 4 | **5** | 6 | 7 | 8 | **9** |  |
| **Rehabilitation services that provide free care or subsidized care for the poor (relative to no such care)** | **1** | 2 | 3 | 4 | **5** | 6 | 7 | 8 | **9** |  |
| **Health insurance coverage for rehabilitation services (relative to no health insurance coverage)** | **1** | 2 | 3 | 4 | **5** | 6 | 7 | 8 | **9** |  |
| **Providing rehabilitation services within specialized hospitals and units (relative to general hospitals or non specialized units)** | **1** | 2 | 3 | 4 | **5** | 6 | 7 | 8 | **9** |  |
| **Having rehabilitation delivered through your health provider (relative to having rehabilitation delivered through other providers /services like social welfare.** | **1** | 2 | 3 | 4 | **5** | 6 | 7 | 8 | **9** |  |
| **Community based rehabilitation (relative to hospital or clinic based rehabilitation)** | **1** | 2 | 3 | 4 | **5** | 6 | 7 | 8 | **9** |  |
| **Multidisciplinary rehabilitation integrated within trauma care (relative to trauma care without rehabilitation services)** | **1** | 2 | 3 | 4 | **5** | 6 | 7 | 8 | **9** |  |
| **The use of data collection / management and dissemination systems (relative to no such use)** | **1** | 2 | 3 | 4 | **5** | 6 | 7 | 8 | **9** |  |
| **Increasing the culture of data collection and use as well as acceptability and reliability of data (relatively to not increasing such a culture)** | **1** | 2 | 3 | 4 | **5** | 6 | 7 | 8 | **9** |  |
| **Provision of assistive technology free of charge (relative to prescription only)** | **1** | 2 | 3 | 4 | **5** | 6 | 7 | 8 | **9** |  |
| **Educational intervention promoting the use of assistive technology (relative to no such intervention)** | **1** | 2 | 3 | 4 | **5** | 6 | 7 | 8 | **9** |  |
| **Tele audiology in comparison (relative to standard face-to-face audiology)** | **1** | 2 | 3 | 4 | **5** | 6 | 7 | 8 | **9** |  |
| **Engaging clinicians / managers to collect and use data (relative to no such engagement)** | **1** | 2 | 3 | 4 | **5** | 6 | 7 | 8 | **9** |  |
| **Home-based rehabilitation programs (relative to usual care)** | **1** | 2 | 3 | 4 | **5** | 6 | 7 | 8 | **9** |  |
| **Tele rehabilitation strategies (relative to usual care)** | **1** | 2 | 3 | 4 | **5** | 6 | 7 | 8 | **9** |  |
| **Task-shifting (relative to usual care)** | **1** | 2 | 3 | 4 | **5** | 6 | 7 | 8 | **9** |  |

**Please give reasons for your rating, paying particular attention to those interventions that have received the highest and lowest scores**

**IV. Closing**

Is there **anything else** that you think is important for improving rehabilitation services for individuals living with disability that has not been mentioned?

**Glossary:**

**Decentralized services** are a complex multifaceted concept where there is a transfer of authority and responsibility for public functions (rehabilitation needs) as a response to the problems of a centralized system (whether private or public).

**Specialty hospitals** are stand-alone, single-specialty facilities not within the walls of a full-service hospital

**Integrated care** is a concept bringing together inputs, delivery, management and organization of services related to diagnosis, treatment, care, rehabilitation and health promotion. Integration is a means to improve services in relation to access, quality, user satisfaction and efficiency.

**Community-based rehabilitation (CBR)** is a multi-sectoral strategy that empowers persons with disabilities to access and benefit from the different sectors within the community (education, employment, health and social services) thus enhancing their quality of life and that of their families. CBR is implemented through the combined efforts of people with disabilities, their families and communities, and relevant government and non-government health, education, vocational, social and other services.

**Multidisciplinary rehabilitation** is rehabilitation delivered by a team of different healthcare professionals (for example, doctors, nurses, therapists) working in an organized manner to address the needs of people living with disability.

**Inpatient Rehabilitation Units** are units that help individuals who have physical or cognitive deficits (e.g. stroke, brain injury, spinal cord injury, orthopedic injury, or those who have a neurological or medically complex condition) to recover from disease or injury and become as independent as possible.

**Interventions to increase the culture of data collection and use as well as acceptability and reliability of data** (e.g. training programs, incentives, feedback on data collection, understanding the importance of data etc.)

**Interventions to engage clinicians/managers to collect and use data** (i.e. incentives, data collection as part of role description, training etc.)

**Tele-health** uses networks to communicate and apply health procedures and treatment plans over long distances to ensure more effective health outcomes**.**

**Tele rehabilitation strategies (relative to) usual care for people with disabilities**: usual care may be for example when we talk about follow-up after discharge – tele-rehabilitation instead of face-to-face follow up or tele-rehabilitation instead of no follow up.

**Task-shifting (relative to) usual care:** Task shifting is a process by which there is delegation of tasks, where appropriate, to less specialized health workers. It presents a viable solution for improving health care coverage by using the already available human resources more efficiently and by quickly increasing capacity while training and retention programmes are expanded

**QUESTIONS FOR PILOTING OF INSTRUMENT:**

1. Is the objective of this survey clear? If unclear, please explain what is not clear.
2. In your opinion, are the objective of this survey and the questions included in this questionnaire relevant? Please explain your answer.
3. Are the terms/definitions included with this questionnaire understandable and clear? Please explain your answer. If unclear, which term is not clear and how should it be changed/clarified.
4. Are the questions included in this questionnaire clear? Please explain your answer. If unclear, please indicate exactly which questions or elements are not clear and how they should be changed/clarified?
5. How was your general experience of this questionnaire? In your opinion, what should be changed to improve the questionnaire? Please give us your suggestions freely.
